# Supplementary material for: Modeling glioblastoma heterogeneity as a dynamic network of cell states
Source: Mol Syst Biol. 2021 Sep 16;17(9):e10105. doi: 10.15252/msb.202010105 (PMC8444284; doi:10.15252/msb.202010105)
Supplement: Supplementary file 6 — Source Data for Figure 5 [file MSB-17-e10105-s004.zip › Figure5A_sourcedata/GSEA_3071/hallmarks_stateIII.GseaPreranked.1623742540963/gsea_report_for_na_pos_1623742540963.html]

Report for na\_pos 1623742540963 [GSEA]

| GS  follow link to MSigDB | GS DETAILS | SIZE | ES | NES | NOM p-val | FDR q-val | FWER p-val | RANK AT MAX | LEADING EDGE || 1 | HALLMARK\_MYC\_TARGETS\_V1 | Details ... | 54 | 0.76 | 7.41 | 0.000 | 0.000 | 0.000 | 420 | tags=83%, list=11%, signal=92% |
| 2 | HALLMARK\_OXIDATIVE\_PHOSPHORYLATION | Details ... | 56 | 0.72 | 6.62 | 0.000 | 0.000 | 0.000 | 429 | tags=82%, list=11%, signal=91% |
| 3 | HALLMARK\_ALLOGRAFT\_REJECTION | Details ... | 36 | 0.23 | 1.78 | 0.000 | 0.020 | 0.055 | 339 | tags=31%, list=9%, signal=33% |
| 4 | HALLMARK\_DNA\_REPAIR | Details ... | 27 | 0.20 | 1.31 | 0.143 | 0.162 | 0.440 | 1116 | tags=56%, list=28%, signal=77% |
| 5 | HALLMARK\_UNFOLDED\_PROTEIN\_RESPONSE | Details ... | 29 | 0.15 | 1.01 | 0.472 | 0.437 | 0.857 | 317 | tags=24%, list=8%, signal=26% |
Table: Gene sets enriched in phenotype **na **[plain text format]****
